# Pathology of the Aorta and Aorta as Homograft

**DOI:** 10.3390/jcdd8070076

**Published:** 2021-06-29

**Authors:** Gaetano Thiene, Cristina Basso, Mila Della Barbera

**Affiliations:** Department of Cardiac, Thoracic, Vascular Sciences and Public Health, Cardiovascular Pathology, University of Padua Medical School, Via A. Gabelli, 61, 35121 Padua, Italy; cristina.basso@unipd.it (C.B.); mila.dellabarbera@unipd.it (M.D.B.)

**Keywords:** aorta, homograft, semilunar valves

## Abstract

The aorta is not a rigid tube, it is an “organ” with lamellar units, consisting of elastic fibers, extracellular matrix and smooth muscle cells in between as parenchyma. Several diseases may occur in the natural history of the aorta, requiring replacement of both semilunar cusps and ascending aorta. They may be congenital defects, such as bicuspid aortic valve and isthmal coarctation with aortopathy; genetically determined, such as Marfan and William syndromes; degenerative diseases, such as atherosclerosis and medial necrosis with aortic dilatation, valve incompetence and dissecting aneurysm; inflammatory diseases such as Takayasu arteritis, syphilis, giant cell and IgM4 aortitis; neoplasms; and trauma. Aortic homografts from cadavers, including both the sinus portion with semilunar cusps and the tubular portion, are surgically employed to replace a native sick ascending aorta. However, the antigenicity of allograft cells, in the lamellar units and interstitial cells in the cusps, is maintained. Thus, an immune reaction may occur, limiting durability. After proper decellularization and 6 months’ implantation in sheep, endogenous cell repopulation was shown to occur in both the valve and aortic wall, including the endothelium, without evidence of inflammation and structural deterioration/calcification in the mid-term. The allograft was transformed into an autograft.

## 1. Introduction

**Figure 1:**
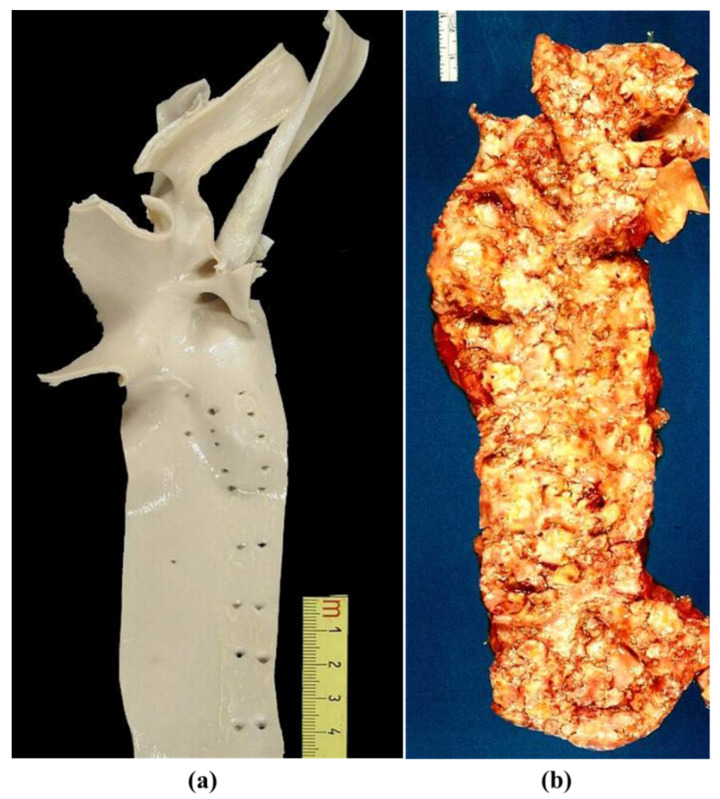
(**a**) Normal thoracic aorta in a child; (**b**) Severe atherosclerosis of the aorta in an old man.

Along with natural history, the aorta, the highway of our body, transforms from a smooth, pliable vessel in children into a rigid tube in the elderly (Figure 1) [1,2]. If normal or nearly normal, it may be donated at the time of death to serve as an arterial allograft for repair of both congenital and acquired diseases.

Here we will review gross and histologic features of normal and diseased aorta. Moreover, we will summarize the results of an experimental investigation, showing endogenous repopulation of decellularized aortic allografts implanted in juvenile sheep.

## 2. Normal Anatomy

**Figure 2:**
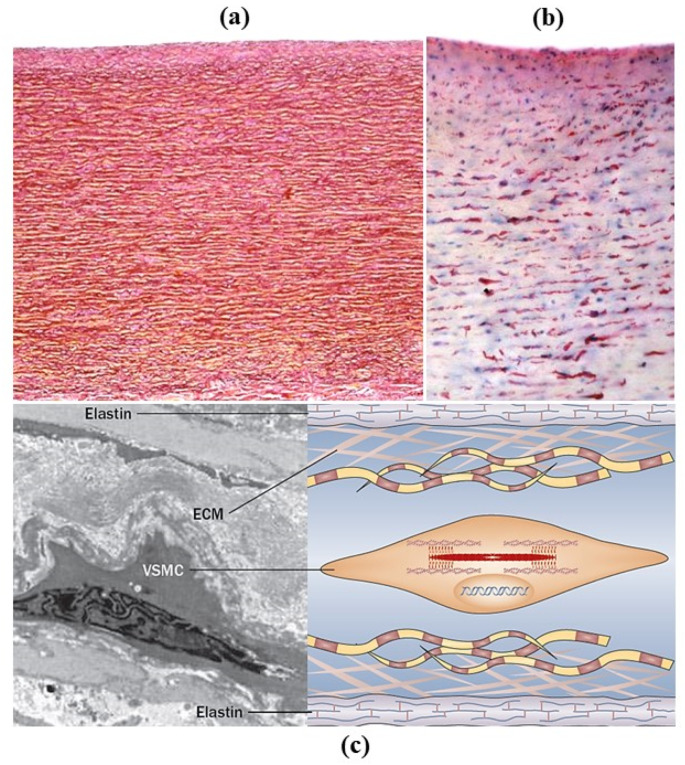
Histology of thoracic aorta. (**a**) The media consists of lamellar units with parallel elastic fibers. Weigert Van Gieson stain. (**b**) Smooth muscle cells, dark stained by anti-SMC immunohistochemistry, are the “parenchyma”. (**c**) The lamellar unit consists of elastic fibers, with vascular smooth muscle cells (VSMCs) and extracellular matrix (ECM) in between. Adapted from [4].

The aorta is a smooth great vessel consisting of intima, media and adventitia. It is an elastic artery with variations in diameter during ventricular systole and diastole (“compliance”), thanks to elastic fibers of the tunica media (Figure 2a). They appear parallel with smooth muscle cells in between (Figure 2b). The number of elastic fibers varies from 56 in the ascending aorta and gradually decreases to 28 in the abdominal aorta. The intima is lined with endothelium and with time appears as a myointimal layer.

There are lamellar units in the media consisting of elastic fibers, smooth muscle cells and extracellular matrix (Figure 2c). The biological concept of the lamellar unit was put forward by Glagov in 1967 [3]. The aorta was considered as an organ with smooth muscle cells as parenchyma. The precise role of smooth muscle cells, whether synthetic, contractile or both, is still a matter under investigation [4,5]. The tunica adventitia consists of collagen fibers, nerves and vasa vasorum. The latter supplies the outer part of the tunica media in the thoracic aorta and not in the abdominal aorta, which is thinner because there are fewer lamellar units.

## 3. Pathology

The pathology may be congenital (structural defect present at birth), genetically determined with onset during childhood or even later, or acquired (inflammatory, degenerative, neoplastic, traumatic).

### 3.1. Congenital Malformations

Congenital malformations may affect the aortic valve, the ascending aorta and the aortic arch [6].

**Figure 3:**
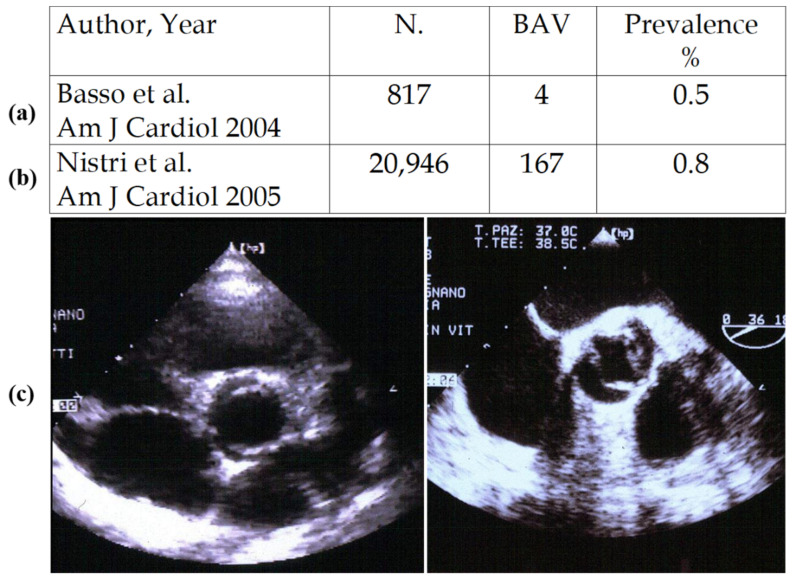
Prevalence of BAV in children (**a**) and conscript soldiers (**b**,**c**) by echo. Adapted from [7,8], with permission.

Bicuspid aortic valve (BAV) is a congenital defect with an incidence of 0.5–0.8% by echo in children (Figure 3) [7,8]. One of the two sinuses shows a raphe, most probably an aborted commissure during embryonic development.

**Figure 4:**
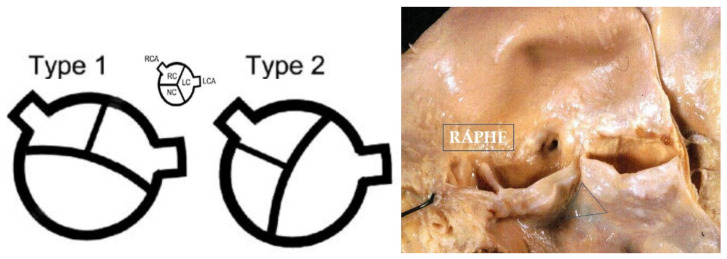
Bicuspid aortic valve, type 1 and 2. Note the raphe without the interleaflet triangle underneath.

In BAV type I the cusps appear antero-posterior (ventro-dorsal) with the raphe in the anterior sinus, due to embryonic fusion of anterior right and left cushions [9]. Both the coronary arteries originate from the anterior (ventral) sinus. In BAV type II the cusps appear side by side (latero-lateral) and the raphe the fusion of right and posterior cushions, with coronary arteries originating each from the opposite sinus. Under the raphe there is no interleaflet triangle (Figure 4). Rare cases of BAV without raphe do exist.

**Figure 5:**
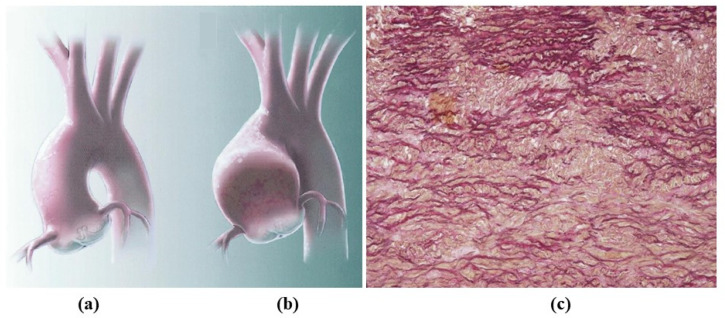
Aortopathy in the bicuspid valve. Note normal (**a**) vs. dilated (**b**) ascending aorta with loss of lamellar units at histology (**c**). (Weigert Van Gieson stain).

**Figure 6:**
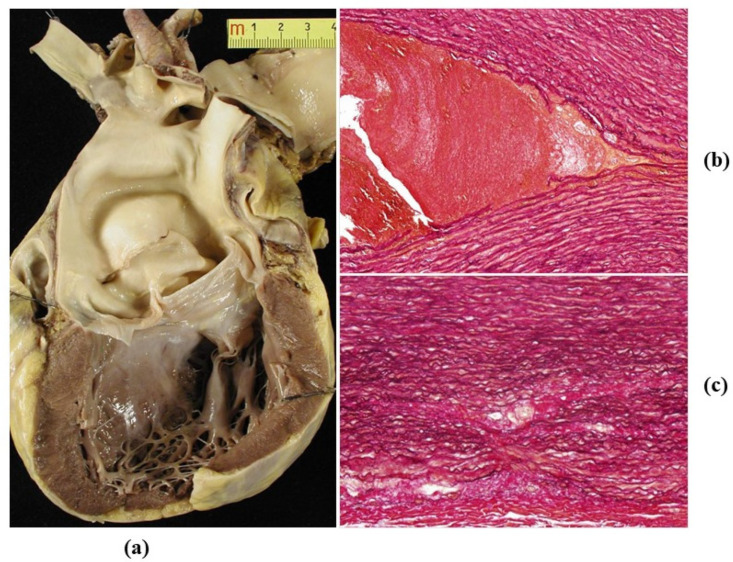
(**a**) Dissection of dilated ascending aorta in the bicuspid aortic valve, with intimal tear just above the commissures. (**b**,**c**) On histology, wave front propagation of the dissecting hematoma and medial necrosis. (Weigert Van Gieson stain).

Natural history of BAV is characterized by progressive calcification of the cusps with aortic valve stenosis [10] and by aortopathy (Figure 5) with dilatation of the ascending aorta, valve incompetence and aortic dissection (Figure 6) [11,12,13].

**Figure 7:**
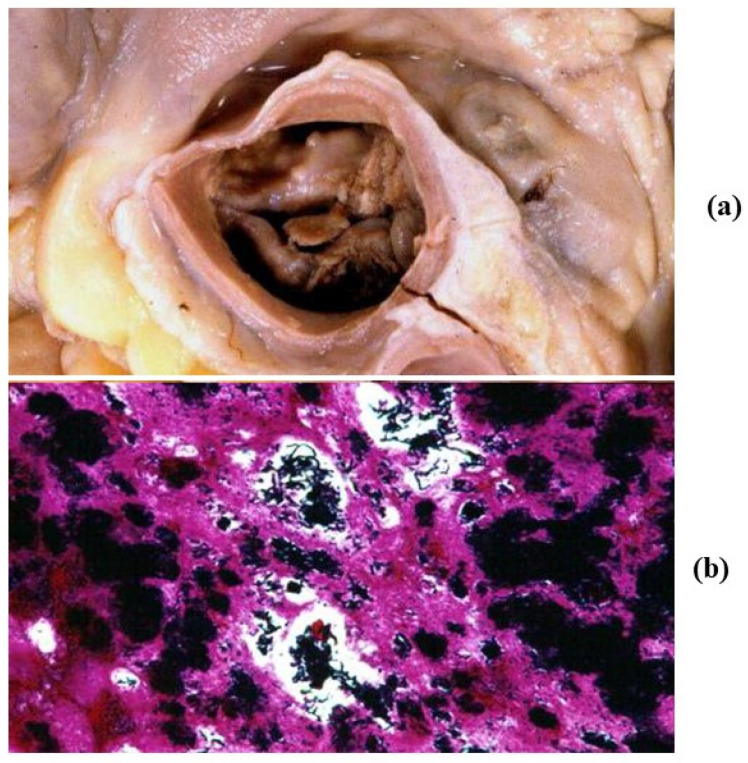
Infective endocarditis (**a**) with cocci (**b**) in a young drug addict with bicuspid aortic valve. (Gram stain).

BAV represents a well-known risk factor for infective endocarditis (Figure 7a,b). It is a cardiovascular risk factor in which antibiotic prophylaxis is recommended, for instance, in case of dental procedures.

Calcification of BAV is similar to senile calcific aortic stenosis and requires interventional repair with transcatheter aortic valve implantation (TAVI) or surgical repair-replacement. It occurs in adults and the elderly, usually ten years earlier than calcific senile.

Aortopathy develops with time in nearly 50% of BAV patients. Disruption of elastic fibers in the tunica media explains loss of wall elasticity as well as aneurysmal dilatation of both sinusal and tubular portions of the ascending aorta, with valve incompetence and even aortic dissection (Figure 6). Surgical replacement of the aorta, with repair of the valve, is indicated when the diameter of the aorta exceeds 5 cm [14]. Both increased aortic diameter and impaired elasticity of the stiffened wall can be assessed by 2D echo [15]. A hereditary Mendelian transmission with scanty penetrance has been advanced, but a definitive molecular genetic background, such as in Marfan syndrome, has not been proven so far.

**Figure 8:**
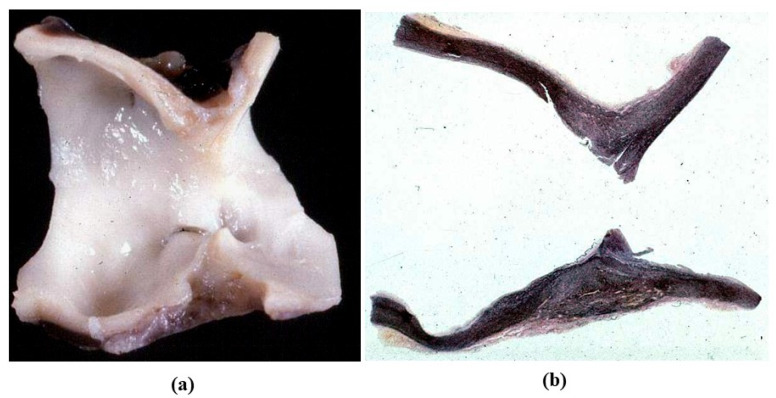
Isthmic aortic coarctation. (**a**) Plication at the aortic arch. (**b**) Corresponding histology. (Weigert Van Gieson stain).

The role of neurocrest in the development of BAV aortopathy has been conjectured since BAV is frequently (40–50%) associated with isthmic coarctation (Figure 8) [16].

Non-infective aneurysm of the sinuses of Valsalva represent a curious congenital malformation of the aortic root [17].

### 3.2. Genetically Determined Diseases of Thoracic Aorta

**Figure 9:**
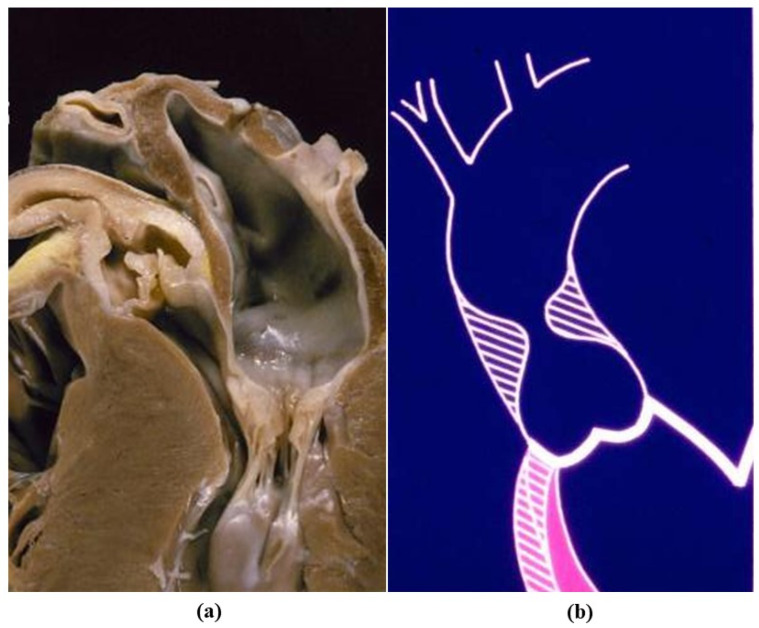
Long-axis (**a**) and hour-glass appearance (**b**) of supravalvular aortic stenosis in William’s syndrome.

William’s syndrome is a dominant hereditary disease, due to molecular deletion of the elastic gene located in chromosome 7, accounting for increased wall thickness and stiffness of the ascending aorta [18,19]. The number of elastic fibers may be increased. Supravalvular aortic stenosis occurs in the shape of an hour-glass (Figure 9) in the diaphragm or hypoplastic ascending aorta. The aortic valve may be also involved with cusp thickening. Insulation of the coronary ostia may be observed because of the fusion of the cusps with sinus aortic wall.

**Figure 10:**
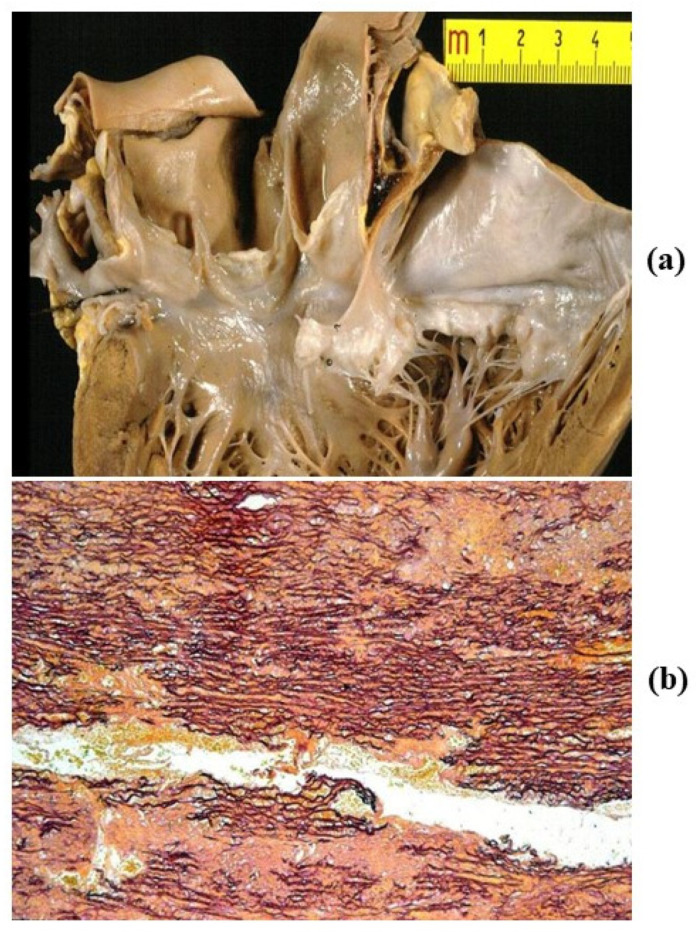
Aortic dissection in Marfan syndrome and sudden death. (**a**) Gross view: intimal tear in the tubular ascending aorta and mitral valve prolapse. (**b**) Histology of the aortic wall: severe elastic fibers frequent in aortic dissection. (Weigert Van Gieson stain).

In Marfan syndrome, the molecular defect consists in a mutation of the gene coding fibrillin 1, a protein connecting smooth muscle cells with elastic fibers of lamellar units in the tunica media [20]. The disease is hereditary dominant, frequently has de novo mutation, and involves joints, lens and the cardiovascular system. It is associated with aortopathy, similar to that of BAV, with disruption and loss of elastic fibers, medial necrosis, aortic dilatation and aortic and mitral valve incompetence. Loss of elastic fibers accounts for weakness and fragility of the aorta to such a degree that aortic dissection may occur, with risk of premature sudden death (Figure 10).

**Figure 11:**
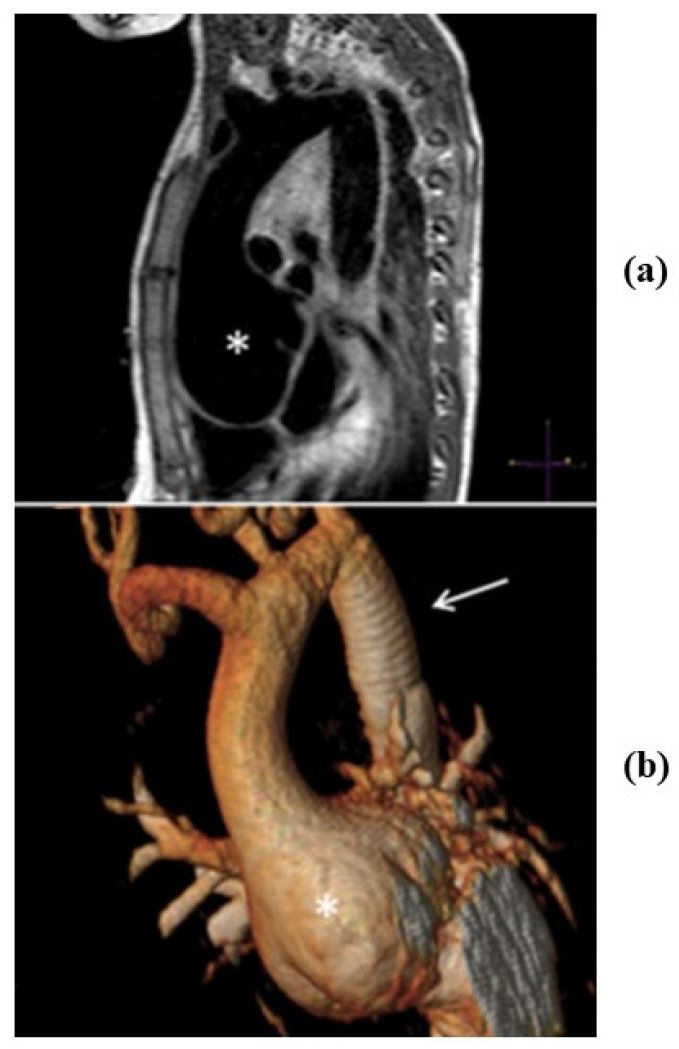
The aorta in Loeys-Dietz syndrome. Notice the large anuloaortic ectasia (**a**,**b**) by tomography. From Rizzo S et al. Ann Thorac Surg. 2016 Mar; 101(3):1193-5, with permission.

Recently, a new syndrome (Loeys-Dietz) has been reported, with an autosomal dominant pattern showing aortic lesions (Figure 11) similar to Marfan. It is due to mutations of genes coding transforming growth factor beta receptor (TGF BR) 1 or 2 [21]. The pathogenetic pathways in Marfan and Loeys-Dietz are similar [22]. Mutations of fibrillin 1 and TGF BR 1 and 2 all result in dysregulated TGB signaling. The main clinical characteristics are hypertelorism, cleft palate and bifid uvula, besides aortic tortuosity prone to dissection.

Ehlers-Danlos syndrome is a soft connective tissue disease, secondary to mutations in one of the genes coding collagen fiber or enzymes involved in collagens synthesis [23]. Six different forms of disease have been recognized. Pathological manifestations in the cardiovascular system are aortic aneurysm and dissection [24].

Turner syndrome is characterized by the absence of one X chromosome in a female. Aneurysmal dilatation of the aorta, BAV and isthmic coarctation are the phenotype, prone to aortic dissection [25].

### 3.3. Degenerative Diseases of the Aorta

**Figure 12:**
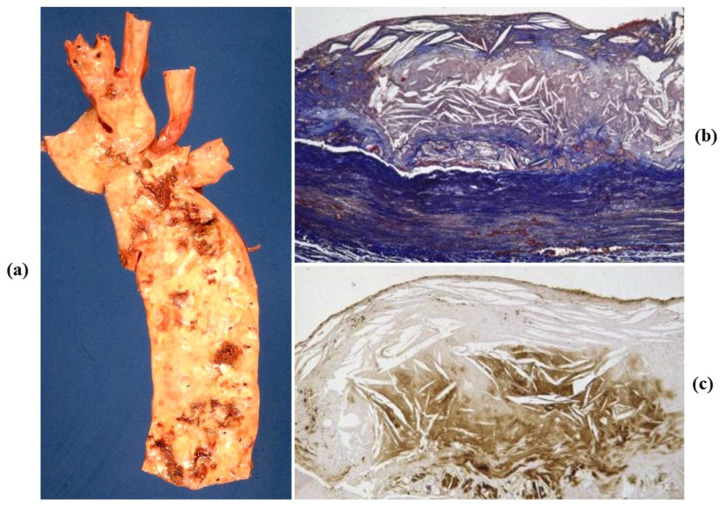
(**a**) Atherothrombosis of the thoracic aorta, gross view. (**b**,**c**) Histology of atherosclerotic plaque. (Weigert Van Gieson and C-68 immuno stains).

**Figure 13:**
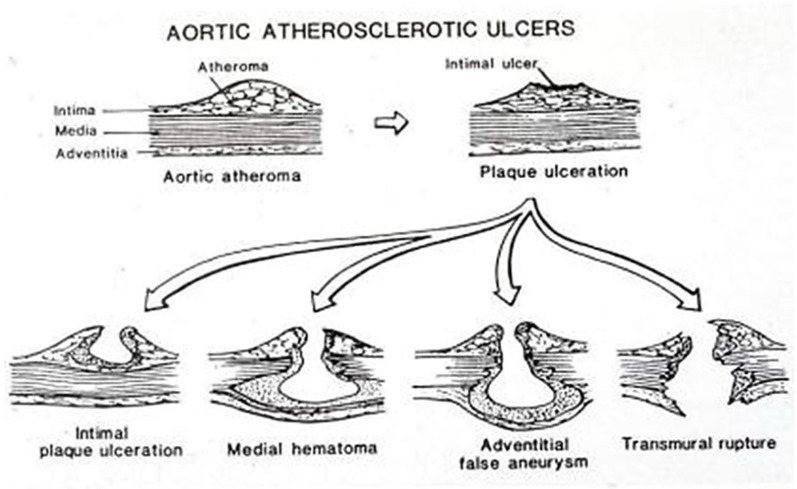
Ulcer of atherosclerotic plaque with intramural hematoma, false aneurysm and external rupture.

Atherosclerosis is the “malignant” disease of the cardiovascular system, mostly related to aging and life style risk factors (smoke, lipid-rich foods…). Premature atherosclerosis is promoted by genetic hypercholesterolemia, the most frequent hereditary cardiovascular disorder (1:500). The disease starts in the intima with atherosclerotic plaque as an elementary lesion, which may complicate with rupture of the fibrous cap and thrombosis (atherothrombosis), a source of peripheral embolism and cause of stroke (Figure 12). The ulcer of an aortic plaque may penetrate into the media and create a local dissection with mural hematoma and false aneurysm at risk of external rupture (Figure 13). Moreover. the ulcerated plaque may be a site of microorganism settlement in the case of bacteremia, with development of mycotic aneurysm.

**Figure 14:**
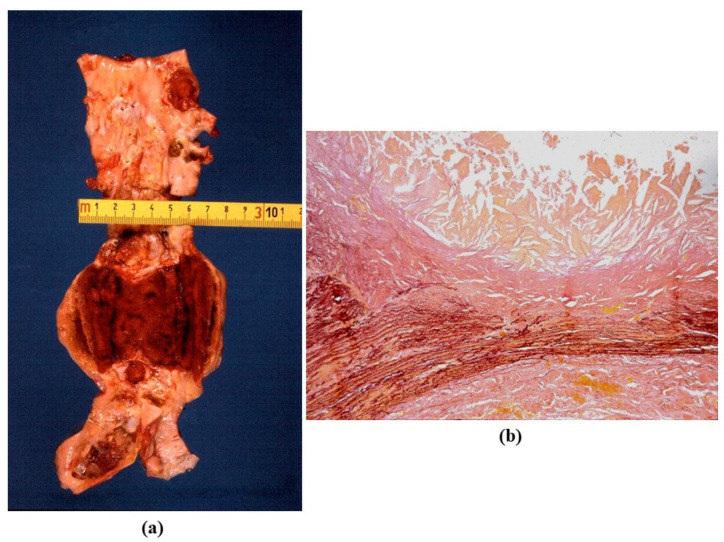
Atherosclerotic saccular aneurysm of the abdominal aorta. (**a**) Gross features. (**b**) Histology with atheroma disrupting the tunica media, with loss of elastic fibers and thinning. (Weigert Van Gieson stain).

Atherosclerotic aneurysm is the complication of progressive thinning of the tunica media due to the release of proteases (elastase included) by monocytes, which play a pivotal role in the onset and progression of atherosclerosis [26]. Release of proteases in the intima may lead to thinning and rupture of the fibrous cap with thrombus deposition. On the outer side of the media, the release accounts for progressive thinning of the aortic wall with aneurysm formation (Figure 14). The occurrence of aortic atherosclerotic aneurysm is more frequent in the abdominal aorta, where the wall is thinner and the number of elastic lamellae is much lower.

**Figure 15:**
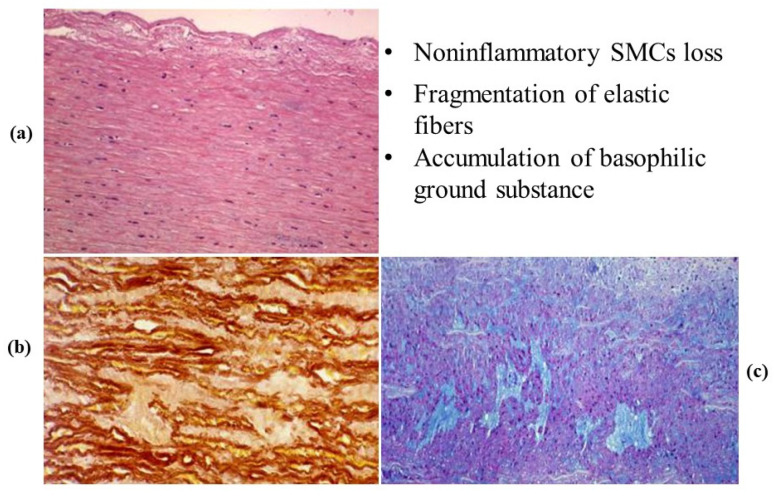
Elementary degenerative lesions in the tunica media of the aorta. (**a**) Non-inflammation loss (medionecrosis) of smooth muscle cells (SMCs). (**b**) Elastic fragmentation. (**c**) Cystic medial necrosis. (Hematoxylin-eosin (**a**), Weigert Van Gieson (**b**), and Alcian PAS (**c**) stains).

**Figure 16:**
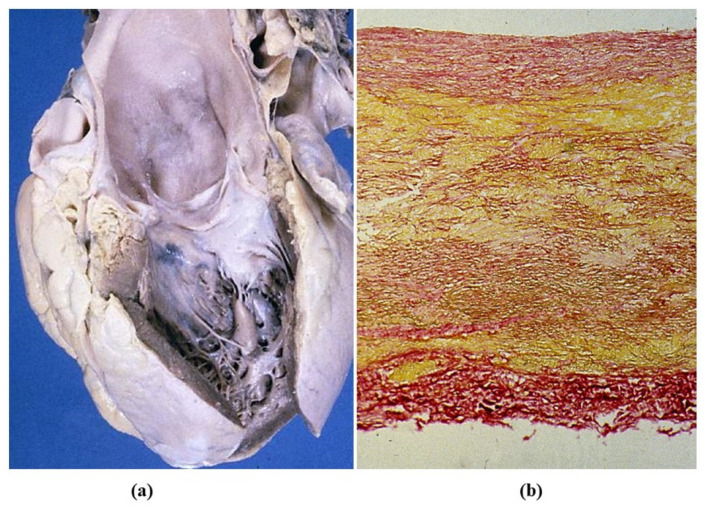
(**a**) Gross view of a dilated ascending aorta with aortic valve incompetence (anuloaortic-ectasia), due to degenerative disease of the aortic tunica media, not genetically determined. (**b**) Severe disruption of the elastic fibers in the tunica media. (Weigert Van Gieson stain).

**Figure 17:**
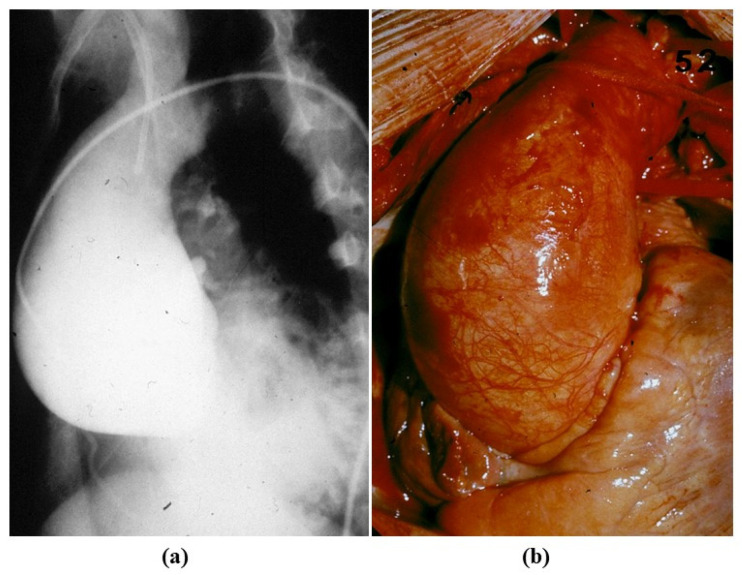
Angiographic (**a**) and intraoperative (**b**) gross views of non-inflammatory anuuloaortic ectasia with valve incompetence.

**Figure 18:**
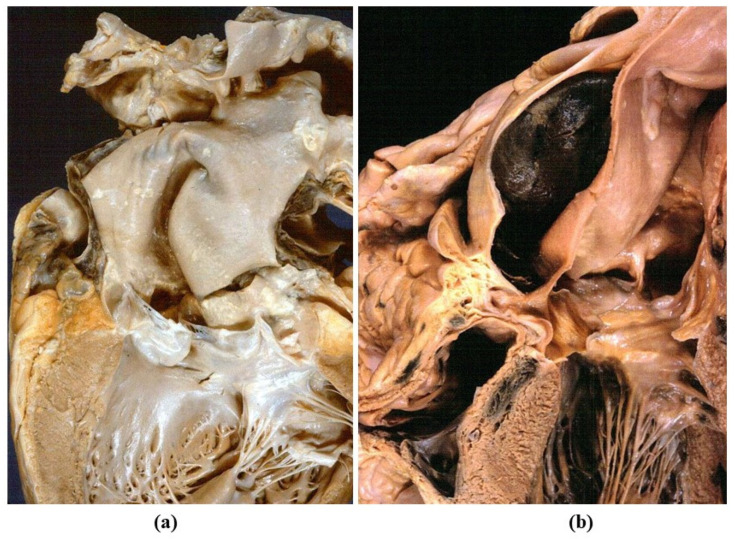
Aortic dissection with intimal tear (**a**) and dissection of the ascending aorta (**b**).

**Figure 19:**
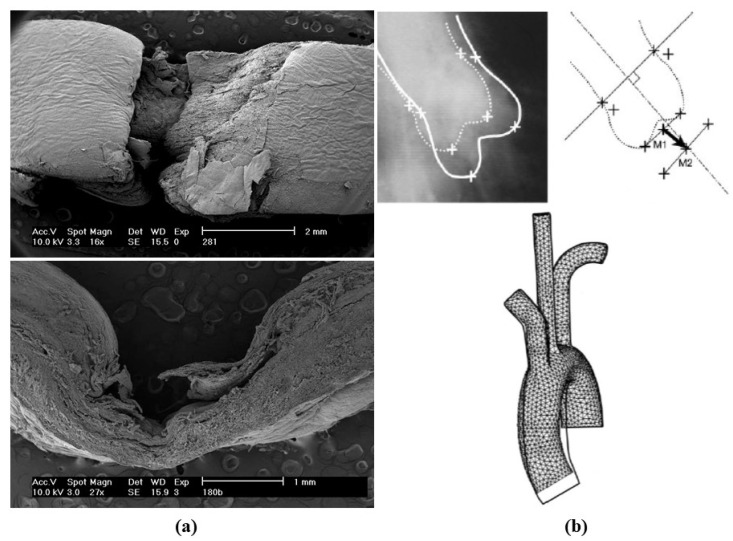
(**a**) Scanning electron microscopy views of an intimal tear in aortic dissection. (**b**) Stretch of the aorta during hypertension attack, accounting for intimal tear and aortic dissection. Adapted from [29], with permission.

**Figure 20:**
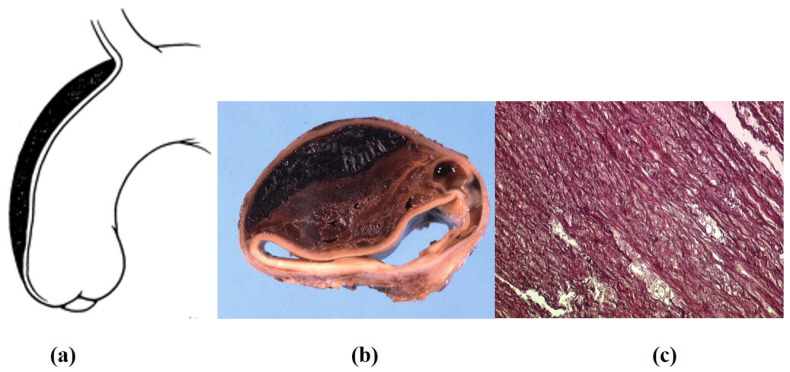
Intramural hematoma in the absence of intimal tear. (**a**) Schematic drawing. (**b**) Gross cross section of the aorta, with intramural hematoma. (**c**) Scattered elastic disruption. (Weigert Van Gieson stain). (**a**) Adapted from [30].

Degenerative disease may primarily affect the tunica media with medionecrosis, elastic fiber fragmentation and an increase in extracellular ground substance in the lamellar units, even in the absence of a genetically determined disorder (Figure 15). It may account for aortic dilatation of the ascending aorta and aortic valve regurgitation, nowadays the most frequent cause of aortic incompetence. Both sinusal [27,28] and tubular portions appear dilated (Figure 16 and Figure 17). It was Currigan of Dublin who first recognized in 1832 the existence of a non-inflammatory cause of aortic incompetence, besides syphilis, rheumatism and infective endocarditis. It represents a fragile substrate favoring aortic dissection, which is featured by intimal tear and dissecting hematoma, triggered by mechanical stress [29] (Figure 18). Hypertensive attack is the main precipitating factor of dissecting aneurysm (Figure 19), which may originate either from the ascending or descending aorta. A more benign form does exist (so-called intramural hematoma), which lacks an intimal tear and is located in the thoracic aorta with the blood source from the vasa vasorum [30] (Figure 20). Hypertension is largely the more frequent risk factor (85%) of aorta dissection [31,32], followed by BAV and Marfan syndrome. Cases have been reported with familiarity (2–3%), distinct from Marfan [33,34,35].

**Figure 21:**
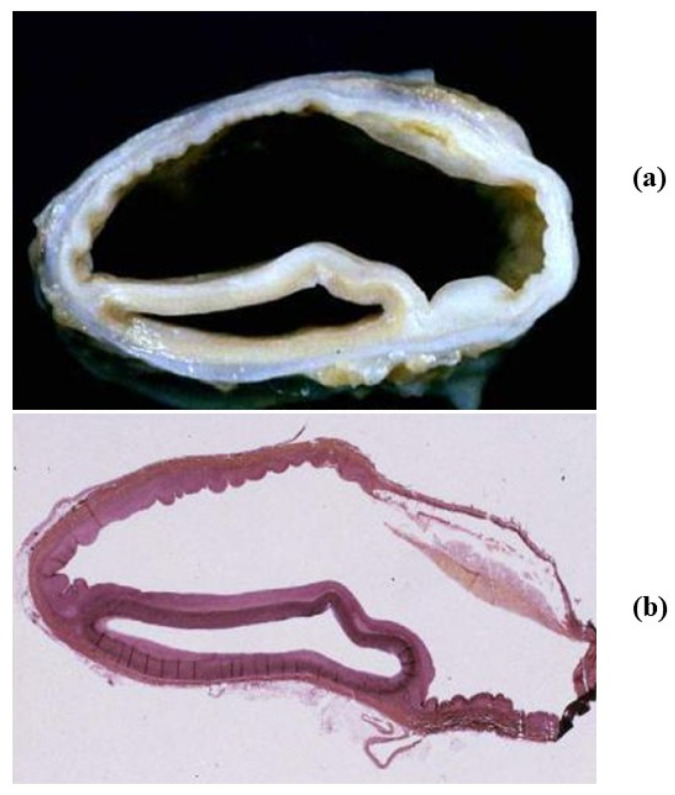
Double barrel aorta in chronic aortic dissection. (**a**) Gross view. (**b**) Corresponding histology. (Weigert Van Gieson stain).

**Figure 22:**
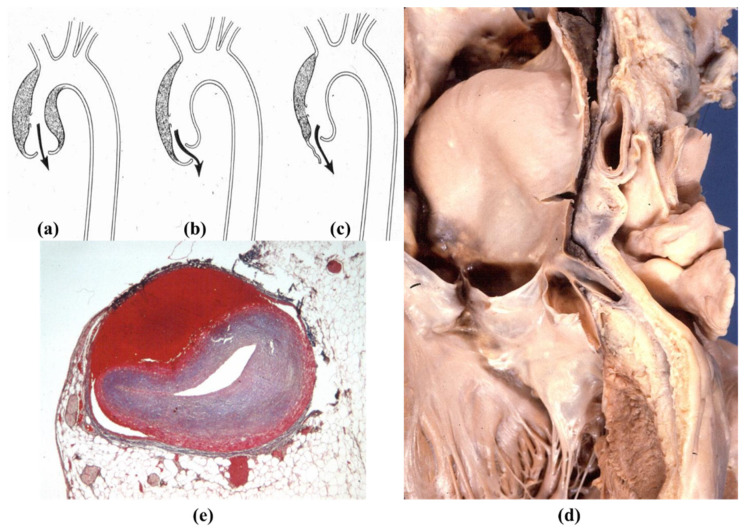
(**a**–**c**) Drawing of aortic incompetence due to retrograde aortic dissection. (**d**,**e**) Retrograde dissection involves and occludes the left coronary artery main stem (Azan Mallory stain).

Classic aortic dissection starts from an intimal tear and proceeds forward (antegrade dissection) in the outer tunica media, with risk of external rupture, hemopericardium and cardiac tamponade in the case of the ascending aorta or left hemothorax in the case of the descending aorta. The dissection transforms the aorta into two lumens (true and false) and may involve vital arteries, such as carotid and renal, with organ ischemic injury. Reentry may occur with distal intimal tears, leading a double barrel chronic dissection of the aorta (Figure 21) [36]. Retrograde dissection towards the aortic root may be responsible for commissural dehiscence with aortic valve incompetence and coronary stems dissection with myocardial infarction. (Figure 22). Hemorrhagic infiltration of the aorto-atrial space and atrial septum may account for av block due to atrio-nodal discontinuity.

### 3.4. Inflammatory Diseases of the Aorta

Inflammatory diseases of the aorta may be infectious or immune-mediated [37,38,39].

**Figure 23:**
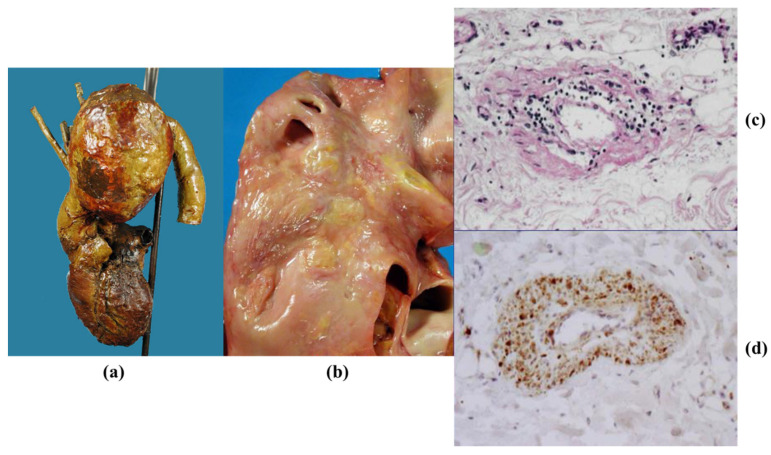
(**a**) Huge saccular syphilitic aneurysm of the aortic arch (from the Morgagni Museum of the Institute of Pathological Anatomy, University of Padua, with permission). (**b**) Syphilitic ascending aorta with pavement-like appearance of the intima. (**c**,**d**) Plasma cell inflammatory infiltrates in the obstructed vasa vasorum. (Hematoxylin-eosin and anti-CD79 immuno stain).

Syphilitic aortitis occurs late in the third stage of the disease [40]. The setting of Treponema pallidum in the tunica media triggers colliquative necrosis (gumma) with giant cells, and disruption of the tunica media (mesoaortitis). The sequela is the development of sacciform aneurysm of the ascending aorta and aortic arch, the rupture of which may precipitate sudden death (Figure 23a). Scarring with retraction of the tunica intima accounts for the specific pavement-like feature and superimposed atherosclerotic plaques (Figure 23b). An alternative theory for mesoaortitis is an obstructive disease of the vasa vasorum of the adventitia with plasma cell infiltrates (Figure 23c,d) causing ischemic damage to the lamellar units of the tunica media. This would explain the absence of syphilitic mesoaortitis in the abdominal aorta, where no vasa vasorum do exist. The mesoaortitis may involve also the sinus portion of the ascending aorta, with valve incompetence, coronary ostia stenosis and angina pectoris.

Infective endocarditis of the aortic valve may involve the annulus, accounting for abscesses and aneurysms in the Valsalva sinus. Optimal repair requires replacement of both valves and the ascending aorta using a homograft [41,42].

**Figure 24:**
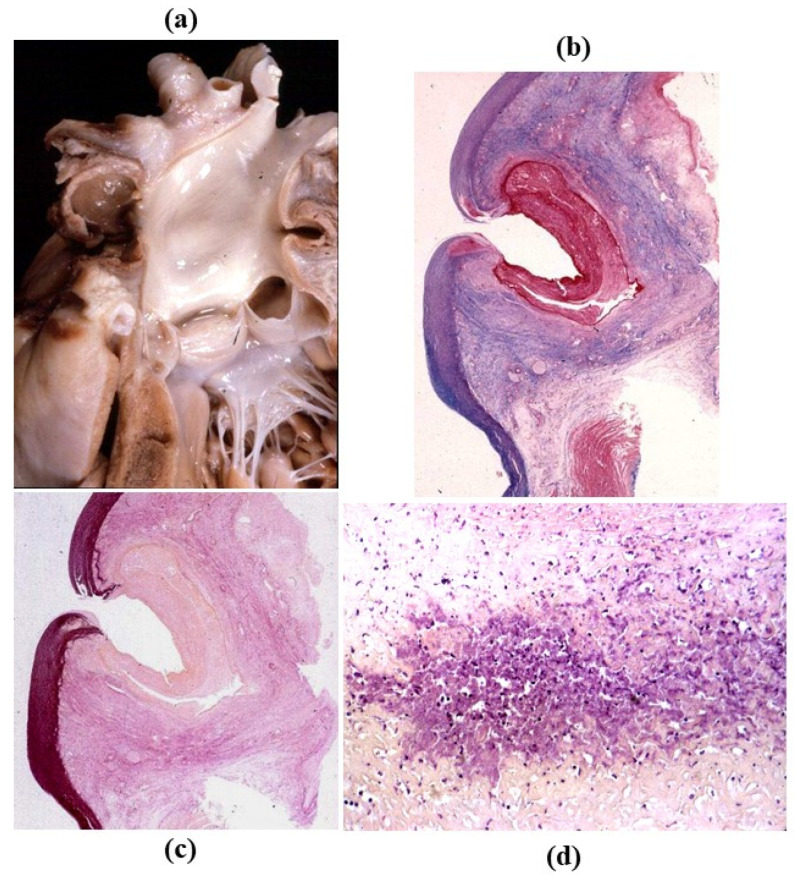
Mycotic aneurysm of the ascending aorta. (**a**) Gross appearance. (**b**) Histology: through and through laceration of the aortic wall with false aneurysm (Alcian PAS stain). (**c**) Same as (**b**) (Weigert Van Gieson stain). (**d**) Abscess by cocci in the aortic wall (hematoxylin-eosin stain).

Mycotic aneurysm is another infectious disease of the aorta. The adjective refers to the look of the aneurysm, similar to a mushroom (or to a nuclear bomb). It develops as the consequence of microorganisms settling in the tunica media, usually cocci bacteriemia via the vasa vasorum, with a neutrophil inflammatory reaction, abscess and transmural necrosis, wall weakening, pseudo-aneurysm and external rupture (Figure 24). Atherosclerotic ulcers are an ideal nidus for bacteria implantation, directly from the aortic blood.

**Figure 25:**
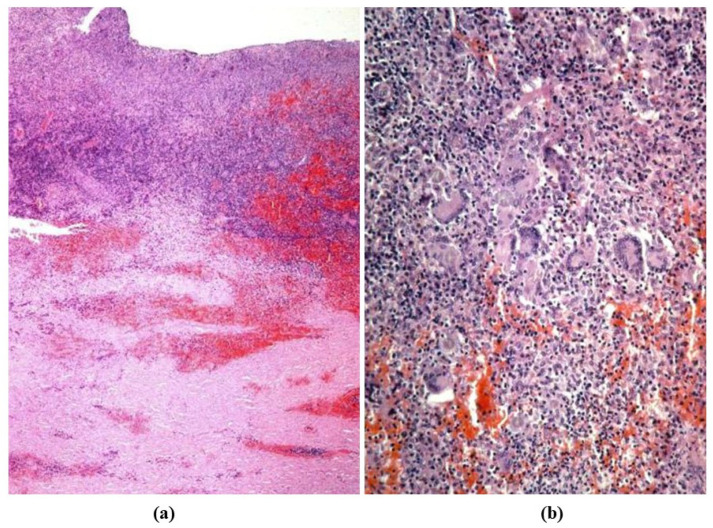
Giant cell aortitis with massive inflammatory disease: (**a**) panoramic view; (**b**) high-power view: note the giant cells (hematoxylin-eosin stain).

Giant cell angiitis involves non-infectious inflammation of the systemic arteries, with giant cell infiltrates in the absence of epithelioid granuloma. It usually affects the temporal artery in adult-elderly people (Horton arteritis). The involvement of the thoracic aorta, whether isolated or in association with the temporal artery, can be complicated with an aneurysm when the tunica media is extensively affected [43] (Figure 25). The diagnosis may come as a surprise at the time of surgical pathology examination since, clinically, it had been considered atherosclerotic.

**Figure 26:**
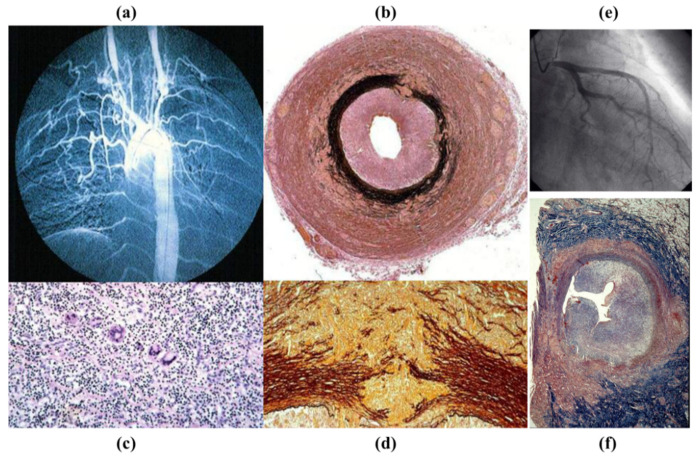
Takayasu arteritis in a 17-year-old girl. (**a**) Angiogram with typical obstruction of the brachiocephalic arteries. (**b**) Cross-section of the left carotid artery: note the severe stenosis of the lumen and the thickness of the adventitia (Weigert Van Gieson stains). (**c**) Giant cell inflammatory infiltrates (hematoxylin-eosin stain). (**d**) Necrotizing angiitis with disruption of the tunica media (close up of (**b**)). (**e**) Left coronary artery stem stenosis by selective coronary angiography with obstructed ostium. (**f**) Corresponding histology (Weigert Van Gieson stain).

Takayasu angiitis is a necrotizing arteritis typically involving the ascending aorta, the aortic arch and brachiocephalic arteries, with non-granuloma giant cells inflammation and elastic fiber disruption [44]. It affects mostly the young and is associated with intimal proliferation of brachiocephalic arteries and coronary stems (Figure 26) as well as fibrotic thickness of the adventitia, hindering aneurysm formation.

**Figure 27:**
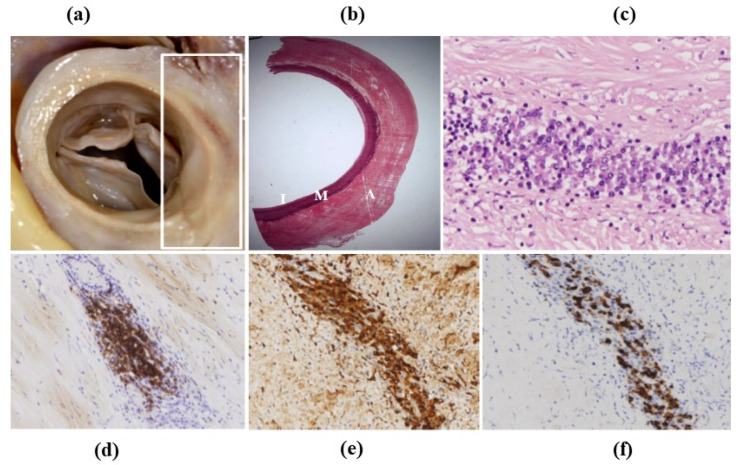
IgG4 aortitis. (**a**) Ascending aorta with increased thickness of the wall. (**b**) Histology: the thickness is located in the adventitia. Weigert Van Gieson stain. (**c**–**f**) Inflammatory infiltrates consist mostly of plasma cells. (**c**) Hematoxylin-Eosin. (**d**) CD79a immunohistochemistry for plasma cells. (**e**) Immunohistochemistry for any IgG. (**f**) Immunohistochemistry for IgG4.

IgG4 aortitis-periaortitis occurs in 8% of IgG4-related systemic arteritis [45]. It involves more frequently the abdominal rather than thoracic aorta and is characterized by wall thickening and inflammatory infiltrates, consisting of IgGM-positive plasma cells and lymphocytes (Figure 27). When the ascending aorta is involved, coronary ostia may become stenotic as to require stenting. 

Ankylosing spondylitis frequently affects the aorta, both thoracic and abdominal, in the form of aortitis [46,47,48,49].

Rheumatoid aortitis [48,50], Reiter’s Syndrome [51], Beçhet’s Disease [52,53,54] and Wegener’s granulomatosis [55,56] represent other systemic morbid entities with inflammatory involvement of the aorta.

### 3.5. Neoplasms

Malignant neoplasms of the aorta may be primary or secondary [57].

**Figure 28:**
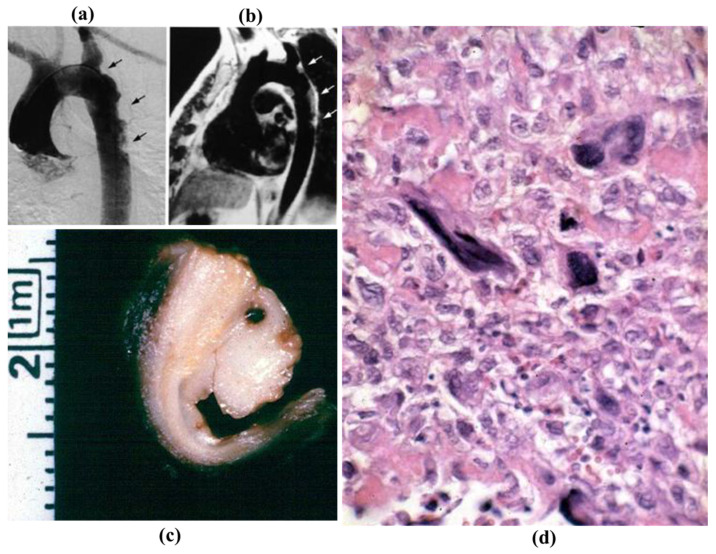
Intimal masses protruding into the lumen of the descending thoracic aorta at selective (**a**) and at tomography angiography (**b**). (**c**) Gross appearance of the specimens removed at surgery. (**d**) Malignant histiocytoma at histology (hematoxylin-eosin).

As far as primary sarcoma, they arise from the intima and appear at clinical imaging as a focal mass protruding into the aortic lumen, mimicking atherothrombosis (Figure 28).

**Figure 29:**
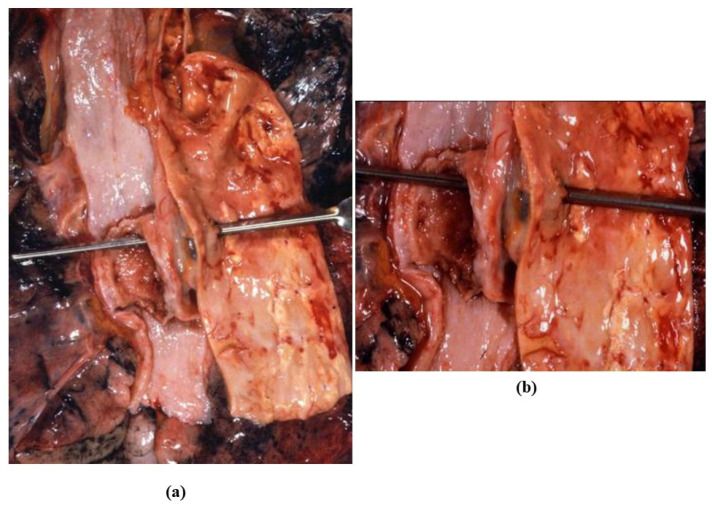
Carcinoma of the esophagus, infiltrating the adjacent aorta and penetrating in the aortic lumen, with massive hematemesis (**a**) Gross view. (**b**) Close up.

As far as secondary malignancies, carcinoma of the esophagus (close to the aorta) may infiltrate and penetrate the aortic wall up to the aortic lumen (Figure 29). Massive gastrointestinal hemorrhage may occur, with blood leaking from the mouth like a fountain.

### 3.6. Thoracic Trauma

**Figure 30:**
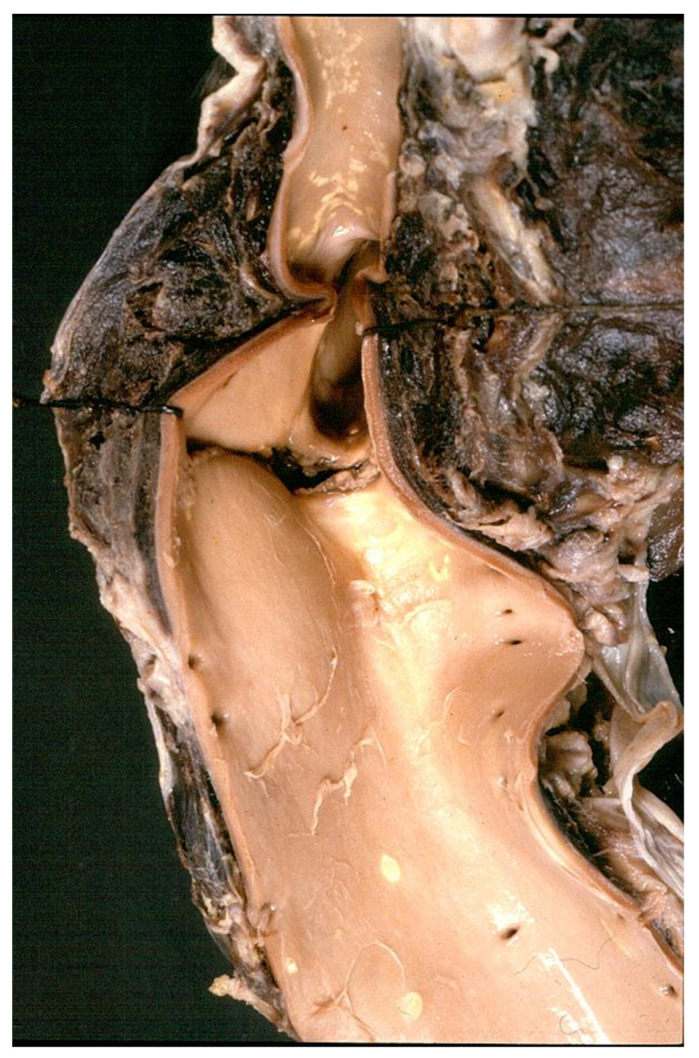
Fatal case of traumatic transmural aortic rupture at isthmic level by blunt trauma.

Penetrating or blunt trauma of the thorax may lead to aortic rupture, usually located in the region of the aortic arch, where the aorta is fixed to the pulmonary artery by the arterial ligament [58]. Traumatic torsion with stretching creates a transmural tear accounting for pseudoaneurysm and external rupture. (Figure 30). An overt aortic dissection is rare.

In conclusion, the aorta is like an organ, with smooth muscle cells of lamellar units as the “parenchyma”. Several diseases may affect the thoracic aorta: congenital, genetically determined, degenerative, inflammatory (both infectious and immune), neoplastic and traumatic. In several morbid entities, the aortic valve, ascending aorta and aortic arch represent an anatomo-pathological unit. Surgical replacement, of both the native aortic valve and ascending aorta, with an aortic homograft is an attractive therapeutic option (see below).

## 4. Aorta as Homograft

Aortic homograft from a cadaver, including both tubular and sinus portions with valves, is surgically employed to replace the native ascending aorta and aortic valve in several of the above-mentioned morbid conditions [59]. However, the ideal graft has not been accomplished so far; the antigenicity of the wall and valve cells triggers an immune cellular reaction.

Tissue engineering techniques can provide a valid approach to solve the problem.

So far, the main common techniques to realize bioengineered grafts are (1) in vitro cell seeding on biodegradable synthetic scaffolds, (2) in vitro cell seeding of decellularized natural scaffolds and (3) in vivo repopulation of decellularized natural scaffolds by circulating endogenous cells [60]. The last choice, however, can be the best to investigate cell repopulation and interaction with the extracellular matrix (ECM), which seems to play a pivotal role in tissue maintenance and regeneration, and modulates cell adhesion and migration, growth factor storage and release, stem cell activation and differentiation [61]. The decellularized homograft appears to be the best scaffold to realize the ideal graft with in vivo self-repopulation [62,63] because it can preserve regeneration capabilities, while the recipient child grows up.

Decellularization of natural scaffold-like aorta (or pulmonary) allografts represents the first step to remove immunogenic cells from both valve and lamellar units [64,65,66].

**Figure 31:**
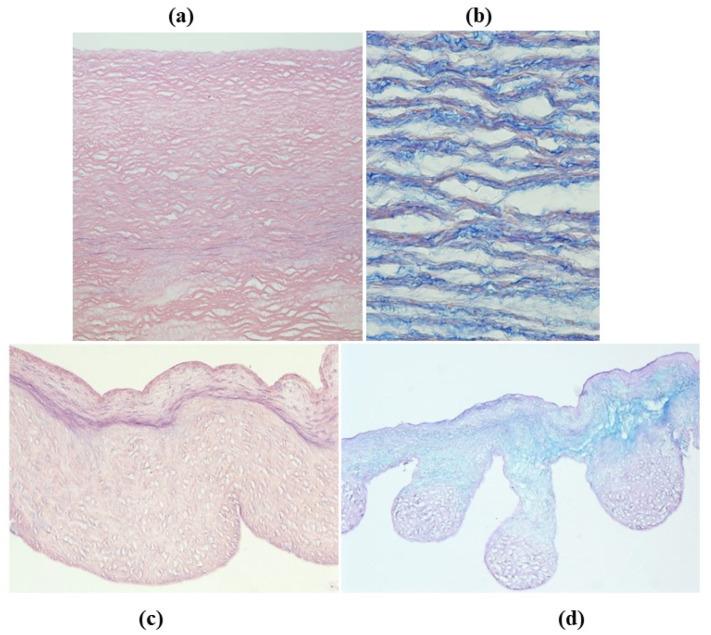
Decellularization of unimplanted aortic homograft: wall (**a**,**b**) and cusps (**c**,**d**). Elastic fibers and ground substance of extracellular matrix are preserved (hematoxylin-eosin, Heidenhein and Alcian PAS stain). From [67], with permission.

**Figure 32:**
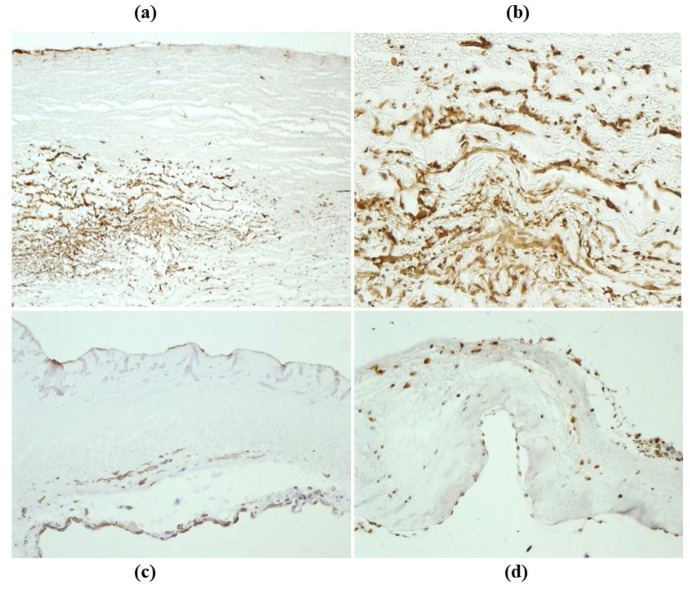
Repopulation by smooth muscle cells of the outer aortic wall (**a**,**b**) with neo endothelial lining (**a**) of a decellularized homograft, 14 months from implant (anti-α-SMA). (**c**,**d**) Repopulation after 14 months of implantation of decellularized homograft aortic cusps by smooth muscle cells and endothelial cells (anti-α-SMA and VWF). From [67], with permission.

**Figure 33:**
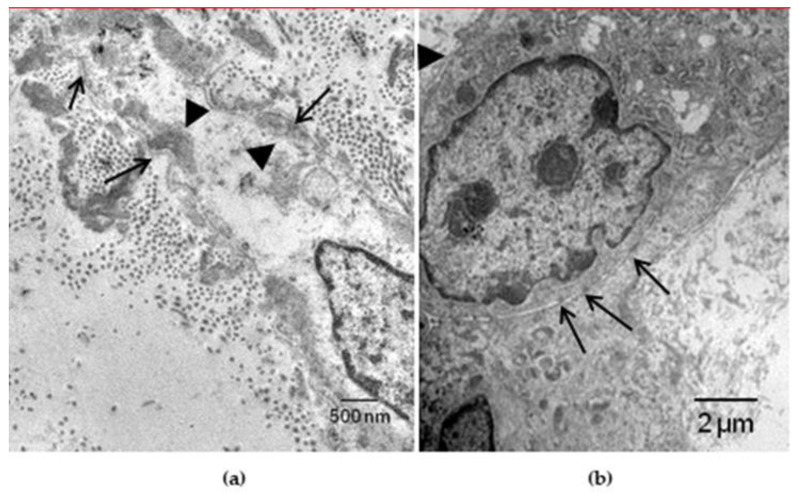
(**a**) Repopulating smooth muscle cells with basal lamina (arrows) and few contractile filaments with focal densities, close to the cytoplasmic membrane and in the paranuclear region (arrowheads). (**b**) Implanted pulmonary homograft cusps: poorly differentiated cells with intercellular junctions (arrows), rough endoplasmic reticulum, and focal basal lamina (arrowhead). From [67].

**Figure 34:**
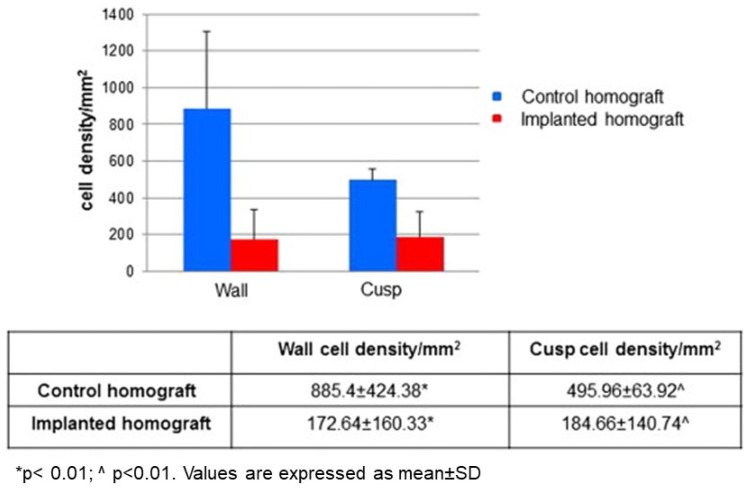
Histogram displaying cell density per millimeter squared in native vs. implanted decellularized conduit wall and cusps. A 20% repopulation occurred. All values are expressed as mean ± S.D. From [67].

With this purpose, we carried out an investigation based on the hypothesis that decellularized homograft may serve as a scaffold for endogenous self-repopulation, thus preventing immune reaction and dystrophic calcification, which is a nightmare with current homografts. The research was carried out in healthy juvenile sheep (35–45 kg) by achieving complete decellularization in unimplanted aortic homograft through 0.5% sodium deoxycholate and 0.5% sodium dodecylsulphate and then implanted in allograft recipients, with a follow up of 14–18.5 months. The morphological study was performed in both aortic cusps and wall, with gross, X-ray, histology, immunohistochemistry, transmission electron microscopy and spectroscopy examinations. Results in detail have been reported elsewhere [67]. In summary:

1. Decellularization in unimplanted allografts appeared complete, both in the lamellar units of the tunica media and valvular interstitium with disappearance of endothelial lining (Figure 31);
2. Cellular repopulation was observed in the outer part of implanted homograft wall by novel smooth muscle cells in the lamellar units (Figure 32a,b) and in the intima with a novel myointimal layer; this layer was noticed also in small animals such as rodents [68]. Novel endothelial cells appeared to line both the aortic wall intima and inflow/outflow at the cusp surface, as well as vasa vasorum, and valve spongiosa appeared repopulated by interstitial cells (Figure 32c,d);
3. The ultrastructure of the wall revealed that novel smooth muscle cells have immature aspects, with a central oval nucleus, few contractile filaments and focal densities mainly located close to cytoplasmic membrane and in the paranuclear region; repopulated cells of the cusps are scarcely differentiated cells, in some case showing short intercellular junctions, rough endoplasmic reticulum and focal basal lamina (Figure 33), whereas others exhibited a fibroblast-like morphology;
4. The undifferentiated nature of the repopulated cells is demonstrated by colocalization of some biomarkers. Novel wall cells showed positivity both for α-SMA and vimentin and novel cusp cells for SMA, vWF, VEGF, VEGF R2, α-SMA and CD57 (HNK-1), which is a neural crest marker;
5. The origin of repopulated cells may be vasa vasorum for the homograft outer wall and the blood stream itself for cusps. Recently it has been demonstrated in a GFP rodent model that all novel cells belong to the recipient [68]. Bone marrow may be a source of progenitor cells (endothelial and mesenchymal cells) contributing to recruitment of smooth muscle-like cells [69,70]. Circulating bone-marrow-derived endogenous cells can be recruited in vivo by adhering to the intimal surface [71,72] and then recruited, undergoing an endothelial-to-mesenchymal transition (EMT) within the valve, followed by differentiation into interstitial cells that ultimately synthesize and remodel the ECM;
6. Cell density, when compared to non-decellularized control allografts, showed 20% repopulation both in the aortic wall and at the cusp level (Figure 34);
7. Mean calcium content by spectroscopy in aortic homografts at 14 months from implant was scanty: 4.24 ± 2.17 mg/g dry weight in the wall vs. 0.530 in controls and 5505 ± 2.04 in the cusps vs. 0.936 in the controls.

In conclusion, cell endogenous repopulation of decellularized homografts occurs at both aortic wall and cusps and persists after implantation, thus providing biological compatibility. An immune inflammatory reaction was not observed. The aortic cusps and wall showed no structural deterioration with negligible mineralization. Decellularized homograft may serve as ideal scaffold for self-repopulation from the recipient, transforming homograft into autograft, even if changes in the ECM constitution were found as compared to native tissue, which could lead to problems in cell growth and migration [63].

These results, however, represent a significant advancement when compared with cryopreserved homografts, which retain allografts cells as a target for immune inflammatory rejection and early calcification.

Finally, investigation of other more specific immunoreactive precursor cell markers is mandatory to study the repopulation cell phenotype and their proliferation modality, especially for the poorly differentiated elements found in the cusps.
